# Fruit Decay to Diseases: Can Induced Resistance and Priming Help?

**DOI:** 10.3390/plants7040077

**Published:** 2018-09-21

**Authors:** Pierre Pétriacq, Ana López, Estrella Luna

**Affiliations:** 1 UMR 1332 Biologie du Fruit et Pathologie, Université de Bordeaux et INRA de Bordeaux, F-33883 Villenave d’Ornon, France; 2 Plateforme Métabolome Bordeaux-MetaboHUB, Centre de Génomique Fonctionnelle Bordeaux, IBVM, Centre INRA Bordeaux, F-33140 Villenave d’Ornon, France; 3 Department of Plant Molecular Genetics, Spanish National Centre for Biotechnology, 28049 Madrid, Spain; ana.lopez@cnb.csic.es; 4 School of Biosciences, University of Birmingham, Edgbaston, Birmingham B15 2TT, UK

**Keywords:** fruit decay, integrated pest management (IPM), post-harvest diseases, induced resistance, priming

## Abstract

Humanity faces the challenge of having to increase food production to feed an exponentially growing world population, while crop diseases reduce yields to levels that we can no longer afford. Besides, a significant amount of waste is produced after fruit harvest. Fruit decay due to diseases at a post-harvest level can claim up to 50% of the total production worldwide. Currently, the most effective means of disease control is the use of pesticides. However, their use post-harvest is extremely limited due to toxicity. The last few decades have witnessed the development of safer methods of disease control post-harvest. They have all been included in programs with the aim of achieving integrated pest (and disease) management (IPM) to reduce pesticide use to a minimum. Unfortunately, these approaches have failed to provide robust solutions. Therefore, it is necessary to develop alternative strategies that would result in effective control. Exploiting the immune capacity of plants has been described as a plausible route to prevent diseases post-harvest. Post-harvest-induced resistance (IR) through the use of safer chemicals from biological origin, biocontrol, and physical means has also been reported. In this review, we summarize the successful activity of these different strategies and explore the mechanisms behind. We further explore the concept of priming, and how its long-lasting and broad-spectrum nature could contribute to fruit resistance.

## 1. Background

Fruit and vegetables represent a major source of food worldwide. They contain a battery of natural compounds with various health benefits, including vitamins, proteins, fibres and minerals. As such, their consumption is highly recommended globally. Quite strikingly, however, more than a third of fruit and vegetables fails to reach the customer due to infections with pathogenic microbes (e.g., bacteria, fungi, viruses) [1]. This leads to massive losses, both economic and social [1]. Diseases caused by such infections are likely to appear in the field (i.e., pre-harvest), but also appear or affect the fruit post-harvest. Post-harvest losses can dramatically impact fruit production and quality [2], resulting in losses of an average of 22.5% of the yield in developing countries [3]. 

However, we live in a world that can no longer afford such high percentages in food waste because, due to an increasing growing population, yields need to rise in order to meet food demands [3]. Importantly, this cannot be done at any cost and should be accomplished in a sustainable manner [3,4]. Currently, the most effective methods of control rely on breeding for resistance and the use of chemical pesticides; however, both strategies are easily overcome by plant pathogens thanks to the evolution against single resistance genes and to chemical targets, respectively [5]. Moreover, the use of chemical pesticides claims further attention due to their potential toxicity to humans and the environment. This is resulting in a growing social demand, forcing the action of public entities for safe and functional food that exploits alternative methods of disease control which could first be used to limit, and then to stop, the use of pesticides [5,6]. In the last few decades, the agri-tech system has put great effort into developing alternatives that could be framed into the so-called integrated pest (and disease) management (IPM) approach.

Among the different control strategies, many research groups are highlighting the potential that exploiting the plant immune system can have in disease protection. Plants benefit from their highly robust and efficient immune system, allowing them to overcome many biological threats. This is due to the fact that, apart from their innate strategies, plants have inducible defence mechanisms to respond effectively against specific threats. Moreover, plants have evolved the capacity to sensitize their immune system for a better expression of induced defence mechanisms [7]. This phenomenon is known as priming of defence and is understood as an adaptive part of induced resistance (IR) [8]. Priming can be first established after stimuli that can have an environmental, biological or chemical origin. After perception, plants maintain a “priming phase” where molecular and biochemical changes occur but where there is not a direct activation of defence mechanisms [9]. Therefore, priming does not result in many costs in terms of plant development [8]. The priming phase has been shown to be long-lasting [10,11] and even to be transmitted to the following generations [12,13,14]. That is, plants exposed to stress stimuli produce progeny that display sensitized defence mechanisms. Upon subsequent attack, priming allows for a faster and stronger activation of defence that ultimately results in broad spectrum disease protection [7]. Therefore, there are many benefits in investigating how plants manage priming: (i) it offers an effective defence strategy against many plant pathogens that are difficult to control by single resistance genes or chemical pesticides [15]; (ii) it results in considerable lower costs in plant growth and yield than direct activation of defence responses [16]; (iii) it has been documented in many plant and crop species [8]; (iv) the expression of the plant’s own defence mechanisms is considered a safer and environmentally friendly approach; and (v) the fact that it is maintained through the life of the plant, including fruit stage [17], opens up possibilities for disease control, both pre- and post-harvest.

In this review, we examine the current knowledge on induced resistance and priming for fruit defence. We highlight the current major threads and methods of control, explore the knowledge in fruit-induced resistance and priming, and examine key aspects of these phenomena that could be combined with current agricultural trends into novel strategies for fruit disease resistance [18].

## 2. Major Post-Harvest Threats to Agricultural Markets

Fruit are threatened by different microorganisms, such as fungi, bacteria and to a lesser extend viruses, which are the agents of different diseases (Table 1).

Post-harvest, the biggest challenge that the agri-tech market faces is definitely of fungal nature. Fungi, without effective control methods, can result in a loss potential of 24% [1]. Most losses of fruit diseases of commercial importance result from pre- or post-harvest infections with fungal pathogens. This is because of their highly adaptive lifestyle that allows them to grow and develop under storage conditions. Major fungal threats post-harvest are moulds, mildews and rots that have the capacity to infect a wide range of plant species.

*Botrytis cinerea* is a necrotrophic fungal pathogen that grows particularly aggressively on the tissues of more than 200 plant species, including fruits and vegetables. *B. cinerea* is the pathogenic agent responsible for grey mould and this disease is capable of affecting many crops at a post-harvest level. Plants belonging to the Solanaceae family such as tomato, and other species of agronomical importance such as strawberries, red fruit (e.g., raspberries), and citrus fruit, are the most affected by this pathogen. Due to this wide range of hosts, and based on economic and scientific importance, *B. cinerea* ranked second into the world top 10 fungal plant pathogens, and is alone responsible for enormous economic losses [19]. Another example of a devastating fungal disease post-harvest is *Penicillium* rots, such as *Penicillium expansum*, *P. digitatum* and *P. italicum*. Similarly to *B. cinerea*, *Penicillium* species cause post-harvest fruit decay in considerable proportions, with *P. digitatum* being responsible for 90% of all the citrus fruit waste post-harvest. Whereas *P. digitatum* and *P. italicum* are responsible for green mould (or green rot) in citrus fruit, *P. expansum* causes blue mould which significantly affects orchard fruit, particularly apples. *P. expansum* is a wound pathogen that uses brushes, punctures or rubs to penetrate in the fruit tissue [20] and spores can live in the soil and organic material (including dead wood). An important difference with *B. cinerea* is that with *P. expansum* serious damage is targeted exclusively at a post-harvest stage. *P. expansum* can be found in vegetative tissue of trees such as citrus and orchards, however, it rarely produces damage that affects fruit production. Nevertheless, as it can be present in the fruit upon collection, methods of control that start before the fruit is harvested are necessary to reduce post-harvest decay to this pathogen. Very importantly, apart from its negative effect in fruit decay, *P. expansum* produces the mycotoxin patulin, a neurotoxic compound that can reach the markets normally in apples and apple products [21]. In addition to these two major mould diseases, mildews such as *Plasmopara viticola* and *Erysiphe necator*, and rots such as *Alternaria alternata*, also result in considerable post-harvest decay of grapes and tomatoes, respectively. Finally, *Colletotrichum* genus fungi trigger anthracnose disease, which is of high importance in many plant species such as bananas, mangoes, papaya and pome fruit. 

In addition to fungi, also bacterial pathogens are responsible for post-harvest fruit decay (Table 1). A vast and diverse community of bacteria can be found on the surface of flesh fruits and vegetables [22]. For example, infections with *Clavibacter michiganensis* subsp. *sepedonicus* cause ring rot in potato and the subsp. *michiganensis* the bacterial wilt and canker of tomato [23], causing important economic costs in certain countries and varieties. It is extremely difficult to control due to bacteria surviving for long periods of time (years) on equipment, storage trays, tools, and other inert surfaces [24]. Other diseases, such as bacterial spot in tomatoes and peppers, are caused by *Xanthomonas axonopodis* and can also result in serious damage to fruit [25]. However, bacterial pathogens seem to have less impact in post-harvest fruit decay in comparison to fungal diseases. For instance, there are crops, such as citrus fruit, that do not develop bacterial post-harvest disease of commercial importance [26]. Nevertheless, it is very important to consider that, apart from their potential effect on fruit decay, fruit can also host bacterial human pathogens, thus representing a serious biosecurity cause of concern. Bacteria such as *Salmonella enterica* and *Escherichia coli* are responsible for human infections. Problems with these bacteria commonly occur in countries where biosecurity and hygienic measures are underdeveloped. However, *S. enterica* infections in tomato occur because of the bacteria accumulating in the fruit after travelling from the rhizosphere. This internal migration is an obscure infection method that is very difficult to control with conventional and hygienic measures. It was only a few years ago when in Germany, an *E. coli* outbreak from infected fenugreek sprouts caused serious damage to human health and the death of at least 55 people [27]. Thus, bacteria of a human pathogenic nature impose a risk in a post-harvest agricultural setting. Currently, there are limited strategies to effectively control them and, therefore, more research is required to limit growth by these strains.

Viruses can also trigger post-harvest fruit decay in different crops, although in comparison to fungal and bacterial diseases, they occur to a much lower extent (Table 1). Typical damage occurs during the growing period in vegetative tissue, thus compromising fruit development and yield rather than affecting the fruit directly. Nevertheless, one problematic virus that results in economic losses post-harvest is the papaya ringspot virus, that affects papaya farming in different countries [28].

There are many threats that challenge production at a post-harvest level. Some of these pathogens, even when causing their biggest damage after fruit harvesting, are known to be already present in the plants during cultivation. Therefore, the implementation of control strategies that target both pre- and post-harvest levels will have the potential to successfully control diseases that claim yield percentages that are not affordable in a world with an ever-increasing population.

## 3. Current Knowledge of Disease Protection

For decades, there have been many methods developed for the control of post-harvest diseases [5]. The use of pesticides [2], biocontrol through antagonistic microbes such as yeast and bacteria [2], the conservation of the microbiome [29], non-toxic agents of plant and other biological origin [30], physical means including ultraviolet (UV) light [31], light-emitting diode (LED) blue light [32], temperature [33], pressure [34], genetic means [35], and the exploitation of the fruit’s own defence capacity [2], have been thoroughly investigated. The development of all these control methods has unfortunately not solved the problem of post-harvest waste to diseases due to the outstanding ability of pathogenic microorganisms to adapt. Pesticide control is arguably the most effective method of control to date. However, pathogens can easily evolve resistance to the active compounds and due to their toxicity to humans and the environment, its use is strictly limited and legislated. Under the directive 2009/128/EC of the European Commission, the use of pesticides at a post-harvest level is highly restricted by maximun residue limits (MRLs), a factor that specifies the safe maximum amount of pesticide that can be found in a particular product. MRLs guide agricultural systems, agri-tech companies and other communities in the design and application of products [6]. This legislation is highly dynamic which makes it complicated to keep up with new requirements. As a consequence, at the moment, there are not many pesticides that can be used at a post-harvest stage. One of them is Imazalil, a product used to prevent fungal pathogens such as *P. digitatum* in citrus and cucurbit fruit [36]. Even if Imazalil is considered safe at present, its use in post-harvest applications is likely to be stopped due to the continued reduction in MRLs permitted. Importantly, there are also some crops, such as tomato and strawberry, where no pesticides can be used to control diseases post-harvest. The agricultural system is therefore trending towards the application of pesticides at earlier states pre-harvest. Pre-harvest treatments when pathogen is still not fully established involves the application of a lower amount of the chemical for the same effectiveness, which in turn reduces chemical residues to comply ultimately with MRLs.

There are several clear advantages of working before the products have been collected: (i) there is a reduction in the mechanical damage that fruit suffer due to manipulation; (ii) it prevents cross-contamination with other diseases during post-harvest treatments; and crucially (iii) it allows for an early intervention when the disease is either still not present in the fruit, or is at the early stages of development. The different pre-harvest strategies are integrated into a battery of measures to ultimately prevent diseases through IPM. This approach consequently provides benefits at both harvest levels and aims to reduce the reliance of crops in pesticide control.

This strategy is not only limited to fungicides, and other approaches are being developed at an earlier stage of cultivation to further prevent post-harvest diseases [37]. For example, other chemicals from plant and other biological origins have proven helpful when applied at a pre-harvest stage [38,39]. Hygienic treatments such as heat and chemical disinfection are regularly followed to combat diseases [40]. They have proven effective in securing that fruit reach storage and distribution in the cleanest possible conditions. Similarly, disease monitoring pre-harvest represents another strategy that provides guidance for specific treatments later on during post-harvest [41]. In addition, the use of biocontrol is a robust and effective method that serves to protect both the plant during growth and the fruit post-harvest [42]. The long-lasting protection is the result of biocontrol cultures being maintained in plants for weeks after inoculation [43], and of the biocontrol initiating its activity at early stages of infection. The long-lasting effectiveness is, however, more complex to deliver under field conditions than in controlled environments. Moreover, post-harvest storage conditions can also impact its performance [44].

Overall, even when the market strongly relies on the use of pesticides, this approach is highly limited and new methods of control have been developed. The novel strategies, however, may lack the expected and needed effectiveness in controlling fruit decay and, therefore, this can trigger distrust in the different partners of the agri-tech market [45]. The direction towards implementing IPM in this system could provide better outcomes to the post-harvest market. However, there is still a lot of work to be done to completely tune the system into providing robust disease control. In addition to the different approaches included in this section, interventions based on the exploitation of the plant and fruit immune system are being developed [2]. They explore the possibility of triggering induced resistance mechanisms in the fruit, which could constitute an additional element in IPM to reduce ultimately the amount of pesticides used in agricultural systems [18]. 

## 4. Induced Resistance for Post-Harvest Disease Protection

In the search for more environment-friendly strategies, many studies have investigated whether the induction of resistance in the host fruit could serve as an effective approach to combat post-harvest waste through diseases. In a recent review publication by Romanazzi and colleagues, it was shown that the number of papers describing induced resistance responses post-harvest have consistently increased through the years, demonstrating the potential of this particular field [2]. In a first study in the early 1990s, Chalutz et al. (1992) demonstrated that the activation of the induced resistance capacity of fruit can represent a method for disease control [46]. This study reported that exposure of citrus to UV light resulted in induced resistance against green mould fungal disease caused by *P. digitatum*.

**Figure 1:**
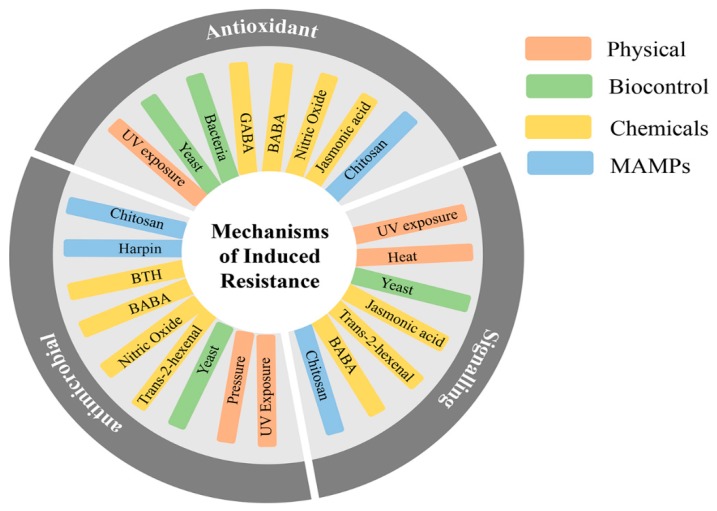
Methods and mechanisms of defence induction. Methods include physical strategies, chemicals, biocontrol and microbe-associated molecular patterns (MAMPs). Mechanisms are classified in signalling functions (accumulation of pathogenesis related (PR) proteins and hormone-dependent signalling), antioxidant functions (reactive oxygen species (ROS) and antioxidant enzymes), and antimicrobial functions (phenolics, lignins and antimicrobial enzymes).

Other methods of defence induction have been reported, including other physical strategies, plant-produced and biological compounds, biocontrol agents and microbe-associated molecular patterns (MAMPs) (Figure 1). The mechanisms by which these strategies trigger induced resistance are very diverse; however, they can be characterized in key groups: (1) accumulation of pathogenesis-related (PR) proteins and hormone-dependent signalling; (2) activation of the antioxidant machinery—reactive oxygen species (ROS) and enzymes such as catalase (CAT), peroxidase (POD), ascorbate peroxidase (APX), and superoxide dismutase (SOD); (3) and antimicrobial enzymatic activity of fruit–phenolic compounds, lignin and enzymes such as chitinases (CHI), glucanases (GLU) and phenylalanine ammonia-lyase (PAL) (Figure 1).

### 4.1. Physical Approach

From that initial paper from Chalutz et al. (1992) other studies have further demonstrated the effect of low-dose UV light in the resistance of fruit to pathogens. They have linked the resistance with the production of antifungal compounds [47] and changes in the activity of defence-related enzymes [48]. In addition to this effective method, other physical approaches have been shown to enhance the resistance capacity of the host. For instance, microarray analysis of heat-exposed peach fruit demonstrated the induction of genes previously involved in resistance such as transcription factors [49]. Moreover, treatment of strawberries with hot air directly triggered the accumulation of PAL, CHI, as well as CAT, APX and SOD, which in turn resulted in the size reduction of lesions caused by *B. cinerea* [50]. In addition, exposing strawberry fruit to hypobaric conditions has also been shown to trigger induced resistance against *B. cinerea* and *Rhizopus stolonifera*, linked to increased CHT, PAL and POD activity [51].

### 4.2. Chemical Approach

The use of chemicals, including fungicides and plant- and biologically-derived compounds, to induce resistance post-harvest has been studied in depth in different pathosystems. Some of these chemicals are plant hormones such as jasmonic acid (JA) and salicylic acid (SA) that are directly responsible for the induction of defence pathways that result in enhanced protection to different diseases. Whereas JA is mostly associated with induced resistance against necrotrophic pathogens, SA is generally involved in mounting defence mechanisms against biotrophic pathogens, through induced systemic resistance (ISR) and systemic acquired resistance (SAR), respectively [52,53]. Importantly, both hormones display in some conditions an antagonistic effect that helps the plant prioritizing a defence strategy over another [54]. The induced resistance effect of a plant hormone in fruit has been studied in depth. For example, the methylated form of JA, MeJA, was shown to be effective in enhancing resistance in peach fruit against *P. expansum*, *B. cinerea* or *R. stolonifera* through increased levels of pathogenic proteins and antimicrobial compounds [55]. Additionally to the fungicidal direct effect [56], treatments with SA have also been proven effective in inducing resistance in fruit against different fungal pathogens, including *P. expansum*, *P. digitatum*, *P. italicum* and *A. alternata*. Antimicrobial and antioxidant enzymes have been involved in the expression of this induced resistance response in many plant species, including citrus fruit, apricots, mangoes and cherries [30,57]. The SA-mimicking agent Benzo (1,2,3)-thiadiazole-7-carbothioic acid S-methyl ester (BTH) has also been shown to trigger induced resistance in melons against *Trichothecium roseum* and mediated by an increased activity of ROS [58]. Also, it has been shown to reduce infection of *T. roseum* in cucurbits such as certain varieties of melon through the activation of the phenylpropanoid pathway [59]. Other plant hormones have also been described to trigger induced resistance in fruit at a post-harvest level. Nitric Oxide (NO) is a gaseous compound associated with the production of reactive oxygen species, is implicated in many different signalling processes in the plant, and has also been shown to play a role in fruit resistance to diseases. For instance, it was reported that external applications of NO could lead to a reduction in citrus fruit anthracnose caused by *C. gloeosporioides* [60]. Its induced resistance activity was totally linked to changes in hydrogen peroxide (H_2_O_2_) levels. Moreover, the accumulation of phenolic compounds and the induction of enzymes such as PAL, POD, CAT and the ascorbate–glutathione cycle were also described to play a role in the induced resistance response.

Other chemicals have also been described to trigger induced resistance post-harvest. Treatments with the chemical volatile trans-2-hexenal triggers induced resistance against *B. cinerea* in tomato fruit that is mediated by the activation of ethylene responsive genes and PAL [61]. Another set of chemicals that have been shown to trigger induced resistance at a post-harvest level are β and γ-amino butyric acid, known as BABA and GABA, respectively [62,63]. BABA is a well-studied non-protein amino acid that primes defence mechanisms through multiple signalling pathways in a wide range of plant species [63]. Recently, its biosynthesis has been demonstrated in various plants, thus promoting BABA to the rank of natural phytohormones [64]. Its effect is better understood in vegetative tissue but it has also been shown that BABA can result in induced resistance post-harvest against different diseases [63]. BABA has been reported to trigger IR through priming against more than 50 biotic and abiotic threats, including fungi, bacteria, herbivory, viruses and drought [63,65]. Importantly, BABA-IR has been proven to be long-lasting in *Arabidopsis thaliana* [11] and tomato [10] and its priming effect was even transmitted to the following generations [13], most likely through changes in the epigenetic machinery of plants. Very recently, we showed that BABA-IR, after application of the chemical to tomato seedlings, was maintained to the fruiting stage providing protection against *B. cinerea* post-harvest [17]. This induced resistance was associated to accumulation of the plant hormone abscisic acid (ABA) in the fruit and highlights a complex role of this plant hormone in BABA-IR. This long-lasting induced resistance was observed in fruit after the treatment of seedlings; however, fruit failed to maintain the resistance phenotype when the treatment had been done once fruit had been produced. GABA is also known for its induced resistance effect, however, in comparison to BABA, the spectrum of action of this chemical is limited [66,67,68]. Nevertheless, GABA treatments seem effective against rot caused by *A. alternata* in tomato fruit. Whereas no direct antifungal effect was observed, GABA was demonstrated to trigger resistance through induction of the antioxidant machinery of the fruit, through the activation of CAT, SOD and POD enzymes.

### 4.3. Biocontrol

Yeast cultures are the most used method of biocontrol of post-harvest diseases against fungal pathogens. Apart from the direct effect in the production of enzymes that degrade pathogen structures [69] and competition for space, different strains have been linked to the activation of resistance mechanisms in fruit. For example, strains such as *Pichia membranefaciens* resulted in the up or down regulation of 25 proteins, which include antioxidants and PR proteins in peach fruit [70]. Similarly, strains of *Aureobasidium pullulans*, and *Cryptococcus laurentii*, induce resistance in cherry tomatoes and peach, respectively, through the activation of host antioxidants metabolism. In addition, induced resistance activity triggered by *A. pullulans* has been linked to the accumulation of GLU, CHI and PAL enzymes [71]. In a lesser extent, other methods of biocontrol based in the use of bacterial strains have been demonstrated to play a role in the activation of induced resistance mechanisms post-harvest. For example, the bacteria *Bacillus cereus* was proven effective to control anthracnose disease caused by *Colletotrichum acutatum* in loquat fruit through induced production of phenolic compounds and H_2_O_2_ [72].

### 4.4. Microbe-Associated Molecular Patterns

In addition to microbiological control measures, it has also been shown that the use of microbe-derived compounds, known as microbe/pathogen-associated molecular patterns (MAMP/PAMPs) can trigger induced resistance responses that ultimately result in protection against post-harvest diseases. This is the case of harpin, a bacterial elicitor that triggers hypersensitive response. Harpin has been shown to induce resistance against *A. alternata* and *Fusarium* spp in melons [73] and black spot caused by *Guignardia citricarpa* in citrus fruit [74]. Treatments with harpin trigger the activation of PAL, 4-Coumarate:CoA ligase and GLU and the accumulation of phenolic compounds and lignin [73]. Moreover, chitin and other derivatives such as chitosan, are also highly integrated within IPM for the protection of post-harvest fungal diseases. Chitin and related elicitors are known to result in a direct effect on the pathogen survival due to their capacity to stop spore germination and/or germ tube elongation [75]. However, chitin and its derivatives are also responsible for inducing resistance through different mechanisms. For example, treatments with the most common derivative chitosan triggered the accumulation of PR proteins, antimicrobial proteins such as GLU and CHI, activation of the antioxidant machinery in fruit through the production of POD, and accumulation of phenolic compounds [75]. The activation of these defence mechanisms has been linked with the induced resistance against many different microbes including *Monilinia fructicola*, *B. cinerea* and *P. italicum* in many different plant species [76,77,78].

## 5. Impacts of Induced Resistance

Induced resistance, therefore, provides many benefits, but further research is necessary to fully integrate its use with other effective control methods within IPM. Importantly, an induction of resistance responses can alter other plant processes. In vegetative tissue, this impact is normally represented by trade-offs in plant growth and development [79]. This is driven by a change in the allocation of energy resources in the plants: when there is an activation of induced resistance mechanisms, plants prevent the allocation of resources to growth as they prioritize the use of energy for their survival against a threat. Other methods of induced resistance also impact plant growth due to other factors. For instance, treatments of high concentrations of BABA in Arabidopsis, as a result of the blocking of the chemical receptor protein (an aspartyl t-RNA synthetase known as IBI1) and the accumulation of the canonical substrate, uncharged tRNA, result in growth reduction [80]. Many groups have investigated the impact of the activation of induced resistance in fruit development and quality. Intuitively, it could be expected that activation of induced resistance would automatically alter fruit growth and quality negatively. However, as seen in this section, many methods of inducing resistance have been correlated with the activation of the antioxidant machinery of the fruit, which could potentially lead to enhanced benefits for human consumption.

Fatemi et al., 2013 for instance, demonstrated that kiwi fruit that harboured increased resistance to grey mould after application of the plant hormone SA, displayed better values in various post-harvest quality factors, including titratable acidity (TA), levels of antioxidants and ascorbic acid (AA) [81]. Similarly, exogenous NO application has been shown to increase AA, TA, and total soluble solids (TSS) content in citrus fruit [60], and treatment with MeJA increases AA content in tomato fruit [82]. In contrast, the application of SA and yeast biocontrol to induce resistance against grey mould did not impact quality properties in peach fruit [83]. In the same lines, treatments of bananas with SA, the SA-mimicking agent BION and K_2_HPO did not result in changes in any of the quality characteristics monitored, including fruit weight, firmness, TSS and TA [84]. On the contrary, treatments with trans-2-hexenal were shown to trigger undesirable changes in the flavour and odour of peach, apricot and nectarine fruit [85]. In addition, we have recently demonstrated that treatments with BABA do have consequences on fruit development and quality by affecting other structural parameters such as fruit size and colour [17]. Tomatoes that came from plants treated with BABA, at the seedling stage and when the fruit had been produced (still green), showed delayed fruit production and ripening. In addition, traces of BABA were found in these tomatoes, which warns of potential risks in commercial applications of this approach [17]. Whether BABA affects chemical fruit content, TA or TSS remains to be elucidated. Nevertheless, it is important to keep these parameters in mind when exploiting induced resistance in fruit, as noticeable changes can hinder the commercialization of the products.

## 6. Towards the Future: Exploiting Priming

### 6.1. Priming for Fruit Resistance

As a more adapted strategy to the expression of defence responses in the fruit via induced resistance, priming of defence mechanisms could provide a solution towards potentially undesirable costs in fruit quality. Primed fruit or plants would not directly activate defence mechanisms unless a challenge is encountered. The costs and benefits of priming with or without second challenge have been extensively documented [16], and studies clearly conclude that upon infection, the benefits that plants obtain from activating primed defence mechanisms outweigh the costs.

Different studies have investigated the possibility that priming in fruit marks induced resistance in fruit post-harvest. For instance, treatments with the chemical GABA in pear fruit result in an induced resistance against *P. expansum* that is based on priming of accumulation of GLU an CHI proteins [66]. Similar priming responses were observed in the induced resistance response triggered by *Candida saitoana* against *B. cinerea* in apple fruit [86]. Moreover, *Bacillus cereus*-mediated induced resistance against *C. acutatum* in loquat fruit is based in priming of expression of defence-related genes (e.g., *NPR1*, *PAL* and *EIN3*) [72]. Thus, induced resistance in fruit can be marked by priming of defence.

### 6.2. Key Aspects for Exploiting Priming in Fruit

**Figure 2:**
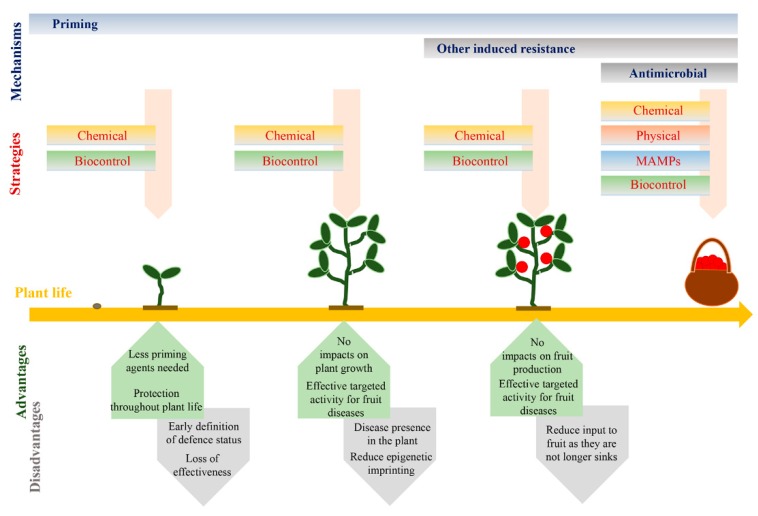
Priming strategies for long-lasting disease resistance in fruit. The described methods of induced resistance are effective from different stages of plant and fruit development. Their expression is based on different mechanisms that can impact the defence capacity of the plant in a short or long-lasting manner. The expression of priming has different advantages and disadvantages depending on when stimuli are applied.

There are different aspects that make priming of defence an appealing phenomenon to study (Figure 2).

Priming is known to result in broad-spectrum resistance due to the target of many different mechanisms. In different systems, priming agents such as BABA and R-β-homoserine (RBH) have been described to trigger induced resistance through priming against diseases of very different natures [87]. In Arabidopsis, RBH was effective against the biotrophic pathogen *Hyaloperonospora arabidopsidis* and the necrotrophic pathogen *Plectosphaerella cucumerina* thanks to the priming of many different defence signalling pathways. In addition, it is easy to identify from the information summarised in Figure 1 that there are induced resistance agents that can activate many different resistance processes. For instance, yeast biocontrol by *A. pullulans* has been shown to trigger GLU, CHI and PAL which in turn results in resistance to pathogens with different lifestyles, such as *B. cinerea* [88] and *P. expansum* [89]. It is likely then, that the priming of those induced resistance mechanisms is behind the protection against all those many different biotic stresses. In addition, priming has also been described to represent a mechanism of enhanced tolerance to drought and temperature stresses [90]. Fruit, as happens with plants during their growing period, can face abiotic stresses. Priming responses have also been linked to enhanced tolerance of peach fruit against chilling injury [91]. Moreover, it was demonstrated that treatments of tomato fruit with SA, for instance, also provides tolerance to cold damage during storage [92]. Therefore, it is plausible that the priming of defence in fruit does also result in protection against abiotic damage triggered by temperature, moisture or mechanical damage due to lack of fruit firmness.

It is also important, however, to take into consideration that there are agents that trigger a specific induced resistance response that is effective against a particular subset of stresses. For instance, trans-2-hexenal results in the activation of ethylene responsive genes and PAL, however, does not induce the accumulation of the PR proteins (e.g., PR-1a and PR-5) [61]. This agent, which leads to the protection against the necrotrophic pathogen *B. cinerea*, could be hindered in the activation of a crucial defence pathway against other pathogens, thus compromising the resistance capacity of the fruit. Moreover, hormone-dependent signalling pathways display antagonistic responses. The best characterised crosstalk of hormone pathways is between SA and JA: the activation of SA-dependent signalling results in the downregulation of the pathways under the control of JA, and vice versa [54]. Therefore, it is plausible that post-harvest induced resistance by agents such as SA, BION or MeJA could result in the downregulation of their counterparts and, therefore, produce fruit that is more susceptible to other specific stresses. Further research is crucial to determine the different responses associated with the use of the priming of defence.

Priming has also been shown to be a long-lasting response [10,11] and even to be transmitted to subsequent generations [12,13,14]. Therefore, priming is a unique form of plant memory. Long-lasting studies support the idea that, similarly to what happens with other control means, an early stimulation during the pre-harvest stage could plausibly provide long-lasting priming that could reach the fruiting stage. This approach would not only benefit from the advantages of early intervention when diseases have not yet fully established themselves, but also could prevent associated costs of induced resistance (Figure 2). Early treatments at a preharvest level with SA and BABA have been shown to reduce disease incidence of *P. digitatum* and *B. cinerea* in orange and tomato fruit, respectively [17,93]. In the case of BABA, the long-lasting induced resistance response correlated with the differential accumulation of metabolites putatively identified as lipids, alkaloids, terpenoids and the plant hormone ABA [17]. This subset of metabolites was speculated to act as a priming fingerprint that marks the better expression of defence mechanisms in fruit. Importantly, however, induced resistance was not observed in fruit when the treatment of plants with BABA was performed after the fruit had been produced, thereby pointing to the lack of chemical relocation to the fruit as they were no longer sinks. However, further research is needed to unravel whether long-lasting SA- and BABA-IR are based on priming of defence mechanisms. Other biological agents are also established at early stages of plant growth, for instance, arbuscular mycorrhizal fungi (AMF). These beneficial fungi have been linked to the activation of priming responses in different crop species, including tomato and wheat [94,95]. However, it is not known whether AMF-induced resistance can reach the fruiting stage and can result in post-harvest protection. Therefore, further research is necessary to address this question, which could foster new strategies based on the use of beneficial microorganisms for long-lasting induced resistance in the fruit.

Interestingly, the long-lasting nature of priming, more commonly understood as a plant memory, has been linked to the activity of epigenetic mechanisms that could fine-tune the expression of defence responses. These studies have been mainly based in Arabidopsis, however, there is evidence that the expression of priming in other crops such as potato and common bean is also linked to epigenetic modifications [96,97]. For instance, long-lasting and transgenerational activity of priming of SA-dependent gene expression relies in changes in chromatin modifications and DNA methylation [11,12]. This brings up the question: can epigenetic changes also be mediating the expression of priming in fruit? It has been previously described that processes such as fruit development and ripening are under the control of chromatin modifications and changes in DNA methylation [98]. Similarly, they have been demonstrated to play certain role in post-harvest processes [99,100]. Considering that fruit are maternal tissue, and having in mind that some pesticides with use pre-harvest can trigger induced resistance and have been linked with epigenetic changes [101], it is very plausible that epigenetic mechanisms play certain role in priming processes in fruit. Actually, changes in the expression of chromatin modification genes have been shown in sweet orange fruit upon infection with *P. digitatum* [102]. The potential of this field is undeniable and it is, therefore, necessary to explore these pathways for disease control. Epigenetic mechanisms could secure an early imprinting of the priming phase towards a faster and stronger disease response in fruit that could contribute to the protection of fruit to post-harvest diseases.

## 7. Concluding Remarks

Overall, induced resistance and priming have emerged as efficient strategies that can contribute to the protection against post-harvest diseases, thereby providing effective alternatives to pesticides for the control of such diseases. Priming-based induced resistance has several benefits: (i) it does not trigger a direct activation of defences therefore does not incur in major costs in growth and development; (ii) it provides broad-spectrum resistance; and (iii) it is long-lasting and can reach the fruiting stage. Nevertheless, induced resistance and priming have also been linked to undesirable effects in fruit quality and other untargeted effects, including susceptibility phenotypes as result of crosstalks in the activation of defence mechanisms. The comprehensive understanding of responses and the adaptation of induced resistance and priming into IPM could provide solutions with a real effect in the protection of fruit post-harvest. With respect to this, the use of computational tools in agricultural research, such as machine learning, would provide a better picture of the multi-layered regulations that occur upon induced resistance [103]. In addition, for instance, the combination of different induced resistance agents has been described in other settings with successful results [79]. Importantly, broad-spectrum priming agents such as BABA do not impact the establishment of beneficial microorganisms such as arbuscular mycorrhizal fungi in tomatoes [10]. Thus, IPM and the provision of plants with different tools that prime their immune system could lead to a successful strategy towards the protection of fruit post-harvest and overall food security for the ever-growing world population.

## Figures and Tables

**Table 1:** Examples of major threats for fruit and the main affected crops.

Pathogenic Microbes	Threats	Crops
**Fungi**	*Botrytis cinerea*	Tomatoes, citrus fruit, grapes, strawberries
	*Penicillium expansum*	Apples, citrus fruit
	*Penicillium digitatum*	Apples, citrus fruit
	*Penicillium italicum*	Citrus fruit
	*Plasmopara viticola*	Grapes
	*Rhizopus stolonifera*	Strawberries
	*Alternaria alternata*	Tomatoes, grapes
	*Fusarium* spp.	Melons
	*Trichothecium roseum*	Cucurbits (e.g., melon)
	*Colletotrichum gloeosporioides*	Citrus fruit, bananas, mangoes, papayas
	*Colletotrichum acutatum*	Loquats
	*Guignardia citricarpa*	Citrus fruit
**Bacteria**	*Clavibacter michiganensis*	Tomatoes
	*Xanthomonas axonopodis*	Tomatoes, peppers
	*Salmonella enterica*	Tomatoes, melons
	*Escherichi coli*	Tomatoes, strawberries
**Viruses**	Ringspot virus	Papayas
